# Lipid stores reveal the state of the coral-algae symbiosis at the single-cell level

**DOI:** 10.1038/s43705-023-00234-8

**Published:** 2023-04-04

**Authors:** Daniel Aagren Nielsen, Katherina Petrou

**Affiliations:** School of Life Sciences, University of Technology Sydney, Ultimo, NSW Australia

**Keywords:** Metabolism, Cellular microbiology

## Abstract

Coral reefs worldwide are threatened by environmental stress. The observable decline in coral cover, is principally due to the intensifying breakdown of the coral symbiosis, a process known as ‘bleaching’. Overproduction of reactive oxygen species (ROS) is considered a key driver of coral bleaching, where environmental stress leads to increased ROS expression. To explore the link between ROS damage and symbiont status, we measured lipid peroxidation (LPO), a ubiquitous form of ROS damage, in the lipid stores of individual endo- and ex-symbiotic algal cells of three coral species, using confocal microscopy and a lipid hydroperoxide sensitive fluorescent dye. We found LPO was higher in endosymbionts, while lipid volume was greater in ex-symbiotic cells. Cluster analysis revealed three metabolic profiles differentiating endosymbiotic (#1: high LPO, low lipid) and ex-symbiotic cells (#3: low LPO, high lipid), with the intermediate group (#2) containing both cell types. Heat stress caused endosymbionts of *Pocillopora acuta* to shift away from cluster #1, suggesting this cluster represents cells in healthy/stable symbiosis. Our study delivers a new means to assess the coral symbiosis, demonstrating that symbiont LPO ratio combined with lipid store volume is a robust metabolic marker for the state of the symbiosis at the cellular level.

## Introduction

Scleractinian corals form the foundation of coral reefs and owe their ecological success to their interaction with single-celled symbiotic algae [1] belonging to the family *Symbiodiniaceae* [2]. The intracellular relationship between the alga and its host has allowed corals to thrive in the nutrient-poor waters of the tropics for over 200 million years [3]. In symbiosis, the cnidarian host provides the alga with reduced nitrogen and other nutrients derived from its heterotrophic lifestyle, while the algal symbiont supports the hosts’ metabolism by providing photosynthetically produced carbon [4–6]. Owing to the complexity of this relationship and, until recently [7], an inability to perform in-vitro experiments on individual functional symbiotic units (i.e. intact host cell with endosymbionts), our knowledge on the fundamental cellular mechanisms sustaining the symbiotic interactions, including those underpinning the selective expulsion of algal symbionts (whether initiated by host or symbiont) from the host tissue [1, 8], is still lacking.

As part of a cost-benefit regulatory mechanism [9], corals control the density of symbionts in the tissue by restricting their growth rate, possibly through nitrogen limitation [10], and by digesting or expelling excess symbiont cells as they grow and divide [1, 11]. However, expulsion of symbionts may also occur in response to unfavourable conditions causing metabolic instability within the symbiont, the host, or both partners. This is evident from the multitude of environmental perturbations that increase the rate of expulsion, including low- [12] or high temperatures and/or high light [13–15], darkness [16], increased availability of dissolved organic carbon such as glucose [17], phosphate limitation [18] and reduced salinity [19]. The excessive loss of symbionts and their photosynthetic pigment from the coral tissue, a process known as ‘bleaching’, is often fatal to the coral as it cannot sustain its metabolism without receiving the energy produced by its symbiotic algae. In recent decades, high temperature-induced bleaching has become a primary driver of decline in reef health and extent across the globe [20]. Notwithstanding established knowledge of the environmental factors that initiate bleaching, little is known about the cellular trigger for the deterioration of the coral symbiosis, nor in which partner the trigger originates.

In eukaryotic cells, metabolic instability during physiological stress can result in increased levels of reactive oxygen species (ROS) within the cell owing to an imbalance between production from metabolic processes and quenching [21]. Unless properly controlled, ROS may cause damage to internal cellular components through oxidation of DNA, proteins and lipids [22], in extreme cases resulting in apoptosis and death of the cell. Due to the often high light and temperature environment of the reef, the photosynthetic machinery is particularly prone to ROS production and damage [23]. For this reason, the role of symbiont photosynthetic stress in the deterioration of the coral symbiosis has long been a primary focus of coral bleaching research [14, 24–26], where excess ROS produced in the heat-damaged photosystem has been proposed to leak into the host cell and initiate the breakdown of the symbiosis [27, 28]. The involvement of ROS in the heat and light-induced bleaching process has been buoyed by numerous studies showing correlative observations of increased cellular ROS damage and/or antioxidants in the symbiont and host with increasing symbiont expulsion [15, 29, 30]. However, recent studies have thrown into question the origin of ROS with evidence pointing to a role of ROS produced in the host cell itself [31, 32]. Importantly, the diversity of responses observed reveals a multitude of possible pathways leading to the breakdown of the symbiosis, which is likely dependent on the resilience of each partner to different stressors.

In the present study, we aimed to investigate the potential role of metabolic instability in the expulsion of endosymbiotic algal cells from the coral tissue. We explored the level of peroxidation of discrete lipid bodies as a measure of excess ROS production and thereby metabolic instability within coral endosymbionts and ex-symbiotic (algal cells inside coral tissue but not encased in a host membrane) cells. Lipid-peroxidation (LPO) and the formation of lipid-hydroperoxides is a common consequence of increased cellular ROS, and for this reason, LPO is considered a sensitive indicator for ROS damage in living cells. We found that the level of peroxidation of lipid bodies was higher in the endosymbiotic algal cells compared to ex-symbionts and that heat stress caused a decline in the proportion of high LPO endosymbionts, suggesting high LPO is a signature of active symbiosis. Based on these findings we argue that the ratio of LPO to lipid accumulation in coral endosymbiotic algae is a strong indicator for the state of the symbiosis and that this metabolic marker can be used to investigate key features of the symbiosis breakdown in future studies.

## Materials and methods

### Coral sampling and maintenance

Fragments of four individual colonies of the branching corals *Pocillopora acuta*, *Porites compressa* and *Montipora capitata* (~15 cm diameter), were collected from the reef flat (spaced at least 10 m apart, ~1 m depth) off Coconut Island (Moku o Loʻe) in Kaneohe Bay, Oahu, Hawaii. The corals were kept (as whole fragments) for the duration of the study in covered (50% shade cloth), outdoor, flow-through tables (volume ~130 L, depth ~15 cm) with continuous supply of bay water (~5 L min^−1^). During the study in May 2018, the temperature of the water in the bay ranged between 23.5 and 25.5 °C (http://www.pacioos.hawaii.edu/weather/obs-mokuoloe/).

### Symbiont extraction and preparation

Individual coral fragments (~2 cm length) were cut from their respective mother colony immediately prior to processing and analysis. To ensure consistency in the physiological condition (i.e. diurnal stage) of the coral colonies, sampling was carried out between 12 P.M. and 2 P.M. All fragments were processed for fluorescence analysis as described previously [33]. Briefly, the coral fragment was placed in a 50 mL falcon tube containing 3 mL of filtered seawater and struck against a hard surface to release gastroderm cells (see ref. [33] and SI for more details on this extraction method). Subsequently, 1.5 mL of the resulting tissue slurry was transferred to an Eppendorf tube and spun down gently (~30 RCF for 30 s), followed by removal of supernatant and resuspension in 1.0 mL of 0.2 µm filtered seawater (FSW). After a second wash as described, the remaining cells were resuspended in 0.5 mL of FSW and fluorescent dye (Image-iT® Lipid Peroxidation Kit, ThermoFisher Scientific, USA) was added to a final concentration of 10 µM. Incubation was carried out in the dark for 20 min in a culture cabinet at 25 °C. Finally, cells were washed twice in FSW to remove excess dye, gently spun down (~30 RCF for 30 s) and resuspended in 50 µL of FSW and immediately analysed on the confocal microscope. Target cells included ‘ex-symbiotic’ algal cells, that is, algal cells extracted from within the coral tissue but not encased in a host cell (i.e. no longer symbiotic) as confirmed by visual inspection, and gastroderm host cells harbouring two endosymbiotic algae (to facilitate visual identification from single ex-symbiotic cells during image processing).

### Retrieval of fully expelled symbionts

To verify that the lipid profiles of ex-symbionts extracted from the coral tissue were equivalent to those of fully expelled algal cells, expelled algal cells from the hard coral *Pocillopora acuta* were obtained through short-term heat treatment of three colonies. Colonies were placed in an aquarium (50 L) positioned inside the flow-through table used for coral maintenance (down-stream of corals being maintained) to ensure similar light field as controls. The water in the treatment tank was continuously replenished with fresh seawater (~1 L min^−1^) and heated to ~30 °C over 3 days (~1.5 °C/day) using two 30 W aquarium heaters. Expelled symbiont cells were collected by placing each coral fragment in 0.4 L plastic beakers containing pre-heated, 0.2 µm filtered seawater. The beakers were floated inside the treatment aquarium for 3 h, after which the coral fragments were removed, and the water was filtered onto individual 5 µm filters using gentle vacuum. Filtered cells were immediately resuspended in 4 mL of filtered seawater and spun down for fluorescent staining as described above.

### Effect of heat stress on endo- and ex-symbionts

The effect of heat stress on LPO of lipid stores in endo- and ex-symbionts was investigated based on data from a separate study also carried out at the Hawaii Institute of Marine Biology (HIMB) and which has been published previously [33]. For the purpose of the present study, the data have been re-analysed using the same methodology as for the new data (see below) and are presented here with details that have not previously been described, and in a new context. A detailed account of sampling, maintenance and imaging methodology for the previous study can be found in Nielsen et al. [33] and the associated SI. Briefly, three colonies of *Pocillopora acuta* were collected from the reef flat (~1 m depth) around Coconut Island in Kaneohe Bay, Oahu, Hawaii and maintained in shaded, flow through mesocosms (vol: 3 m^3^, flow: ~180 L h^-1^, light: mid-day max ~400 µmol photons m^−2^ s^−1^, natural day-night light cycle). One set of coral fragments was cooled to maintain temperatures below 27 °C, while a second set was subjected to daily warming (naturally fluctuating day-night cycle from 27 °C up to 31 °C), which over the three weeks of incubation resulted in ~50% reduction in tissue symbiont density (bleaching). Incubation, sampling and analysis of cells were carried out as described for the present study.

### Confocal microscopy

The ratio-metric fluorescent dye Image-IT™ based on the BODIPY® 581/591 C11 reagent was used to detect the peroxidation state of lipid bodies within the algal cells. According to the manufacturer, the reagent localises to all lipid membranes and upon oxidation by lipid hydroperoxides displays a shift in peak fluorescence emission (reduced lipids ex/em: 581/590, oxidised lipids ex/em: 488/510). The dye binds strongly within the cell, is pH insensitive and very light stable [34]. Stained cells were analysed using confocal fluorescence microscopy (LSM 710, Zeiss, Oberkochen, Germany) equipped with a temperature controlled environmental chamber (Incubator Xl S Examiner, Zeiss, Oberkochen, Germany), as detailed previously [33]. Endosymbiotic gastroderm cells containing two algal symbionts (for easy identification) and ex-symbionts (non-symbiotic but still within coral tissue) were located visually under bright field and subsequently imaged at 630X (Zeiss Plan-apochromat 63×/1.40 Oil DIC M27 lens). Fluorescence imaging was carried out using 561 nm and 488 nm lasers for excitation of reduced and oxidised lipid, respectively, with fixed collection ranges and laser intensities (pinhole size: 1.51 AU, Image resolution: 512 × 512 px [135 × 135 μm], pixel dwell 1.58 μs, no averaging, z-thickness ~1.0 μm.). For fluorescent stains, exposure time was adjusted to minimise auto-fluorescence in any of the fluorescent channels using unstained control cells. In all cases, auto-fluorescence was negligible compared to the intensity of the fluorescent stain.

### Image analysis

Detection, quantification and fluorescence measurements of lipid bodies within each cell were carried out using a custom macro in ImageJ/Fiji [35, 36] (for detailed description of method see SI). All extracted image data were analysed using R [37]. The fluorescence ratio (oxidised/reduced) of each lipid body was calculated and the average fluorescence ratio of all lipid bodies within each cell was used for further analyses. By using a ratiometric approach, any potential bias in the measurements from variation in dye concentration within the cell or lipid body was eliminated. An estimate of total lipid body volume per cell was generated as the sum of the area of all lipid body ROIs within a cell multiplied by the image layer focal thickness (~1 µm). For endosymbiotic cells, the average of the two cells within one gastroderm cell are presented as one measurement. The large, round and highly fluorescent inclusion bodies sometimes found in algal symbiont cells were excluded from the dataset using fixed size range and fluorescence ratio cut-offs based on manual curation.

### Statistical analyses

All analyses were performed using R Statistical Software (v4.1.2; R Core Team 2021). As the focus of this study was to understand physiological changes between cell types within species of corals, to better compare the patterns across species, all data were normalised to the mean of the value of the respective endosymbiotic cells for each species. Data for comparison were checked for normality and homoscedasticity using Shapiro-Wilk and Levene’s test, respectively. Data that did not meet the assumptions were log or square-root transformed and normal distribution verified using qqplot before running statistical tests. Differences between cell types were analysed with linear mixed effect models on mean colony values and colony as a random factor using the lmer function from the R package ‘lme4’ [38], with parameters estimated using restricted maximum likelihood (REML). For analyses including multiple groups, ANOVA tables for fixed-effects terms with Satterthwaite’s method for denominator degrees-of-freedom and F-statistic were generated using the anova function in R package lmerTest [39]. Data were considered significant at *P* < 0.05. Cluster-analysis was performed using the K-means algorithm (Euclidean distance) from the R package ‘cluster’ [40]. The optimal number of clusters were evaluated using ‘Total Within Sum of Squares’, ‘silhouette’ and ‘gap statistic’ with 100 Monte Carlo bootstrap samples using the fviz_nbclust function from the R package ‘factoextra’ [41].

## Results and discussion

The highly lipophilic and ratiometric nature of the lipid peroxidation sensitive dye employed [34] meant that, using fluorescence confocal microscopy, we were able to measure the level of peroxidation as well as assess the total volume of lipid stores (oxidised and reduced, combined) within individual algal cells. While other lipid membranes may be closer to the origin of the production of ROS and therefore more prone to peroxidation than lipid storage bodies, such as those in the thylakoids of the chloroplasts, we chose to focus on lipid bodies for assessing LPO due to their spatially discrete nature and a strong signal from the fluorescent dye, allowing for clear delineation during image processing and thereby high sensitivity in our measurements.

### Lipids accumulate in ex-symbiotic state

Similar to the results of our previous study on the hard coral *Acropora millepora* [42], we found that on average, ex-symbiotic cells (i.e. non-symbiotic) contained greater amounts of lipid (as lipid bodies) compared to their endosymbiotic counterparts (*M. capitata*: *T*(4) = 2.97, *P* = 0.041; *P. compressa*: T(2) = −20.74, *P* = 0.0023; *P. acuta*: *T*(4) = −6.32, *P* = 0.0032). While only host cells with two endosymbionts were included for analysis in this study, endosymbionts within single-symbiont host cells displayed a similar lipid profile to double endosymbiotic cells (Supplementary Fig. S1 and associated results), verifying that the observed low lipid content in endosymbionts in the presented data was not a result of recent division of the algal cells *in-hospite* and that the cells we termed ‘ex-symbionts’ were not algal cells mechanically broken out of their host cell during processing. To further verify that the ex-symbiotic cells were representative of cells expelled from the coral colony, we compared the lipid profiles of ex-symbiotic cells with expelled cells obtained from the same colonies of *Pocillopora acuta* (Supplementary Fig. S2 and associated results). These data confirmed that the lipid profile of the ex-symbionts was similar, both in terms of lipid content and LPO ratio, to that of cells that had been expelled from the host coral. Lastly, we found a strong negative correlation between lipid content and symbiosome membrane fluorescence (R^2^ = 0.37, *P* < 0.0001, Supplementary Fig. S3 and associated results), verifying that the loss of the symbiotic relationship is correlated with increased lipid content. From these data, we are confident that ex-symbiotic algal cells found within the coral tissue are algae in a post-symbiotic state and in the process of being expelled from the coral colony.

### Lipid body peroxidation is higher in endosymbiosis

**Fig. 1:**
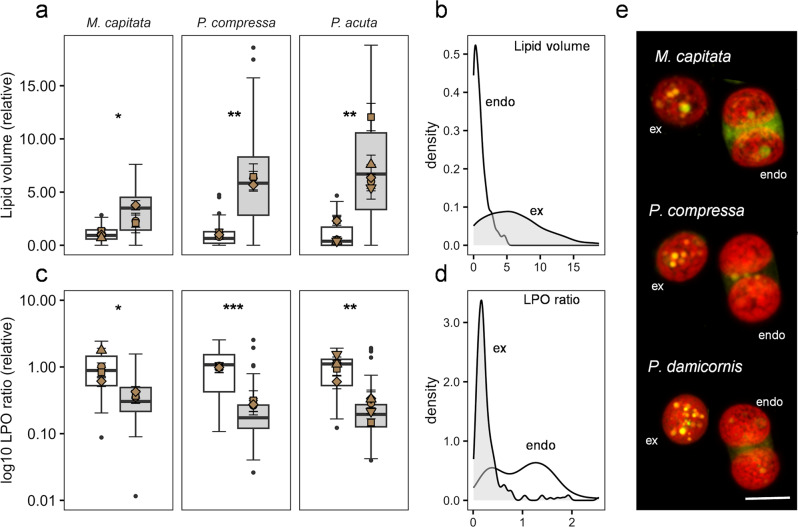
Boxplots and density graphs of lipid volume and lipid peroxidation (LPO) ratio of endosymbionts (white) and non-host associated (ex-symbiotic) algal cells (grey) of three species of hard corals (*M. capitata: Montipora capitata, P. compressa: Porites compressa, P. acuta: Pocillopora acuta*). **a** lipid volume relative to the mean of their respective endosymbionts, overlaid with mean colony value (brown shapes). **b** density graph of lipid volume of all endosymbiotic (white area) and ex-symbiotic cells (grey area), respectively. **c** Lipid peroxidation ratio relative to the mean of their respective endosymbionts, overlaid with mean colony value. **d** density graph of lipid peroxidation ratio of all endosymbiotic (white area) and ex-symbiotic cells (grey area), respectively. **e** sample images (maximum projection of confocal image stacks) of endo- and ex-symbionts from each species. White line indicates size ~10 µm. Red: chlorophyll autofluorescence of algal cells; yellow: lipid bodies with low LPO (green/yellow) ratio. Error bars indicate 1 SE. Stars indicate significance level for comparison of groups (*n* = 3–5) (* *P* < 0.05; ** *P* < 0.01; *** *P* < 0.001).

Contrary to expectations, we found that under conditions free from environmental stress, endosymbiotic algal cells exhibited higher levels of LPO in their lipid bodies (presented as the ratio of peroxidised to non-peroxidised lipid) than ex-symbiotic cells. This difference was consistent in all three species: *M. capitata* (T(2) =−4.852, *P* = 0.040), *P. compressa* (T(2) = −43.391, *P* = 0.00053) and *P. acuta* (T(2) = −5.972, *P* = 0.0094) (Fig. 1c, d), confirming that this pattern is conserved across a range of phylogenetically distinct coral species. These data suggest that endosymbiotic algal cells experience higher ROS pressure per lipid body than those that have been or are being expelled from the host. At a given level of ROS pressure (similar metabolic activity or stress), we would expect a cell with lower lipid stores to become more oxidised per lipid molecule compared to a cell with larger lipid stores, purely as a result of a higher ROS to lipid ratio, which may explain, at least in part, the difference in LPO between symbiont status observed here. However, when comparing the relationship between lipid volume and LPO ratios within cell types using generalised least squares models, the best-scoring model (lowest BIC) included both lipid volume and cell type as predictor variables (see supplementary table S3), indicating that the relationship between lipid content and LPO ratio were different in the two cell types, with endosymbiotic cells exhibiting higher LPO at similar lipid volume (supplementary Fig. S4 and associated results). Based on these results, and given that these data were obtained under benign environmental conditions, we propose that the higher level of LPO observed in the endosymbionts is not a result of stress-related ROS production but rather of ROS produced as part of the general metabolic activity of the cell (respiration and photosynthesis)—which would likely be higher *in-symbio* as the algal cell works to satisfy the metabolic demand of its host cell. This supposition is supported by our previous work where heat stress was shown to reduce the photosynthetic capacity of endosymbiotic algae in the hard coral *Acropora millepora* [42] and reduced the quantity of proteins linked to energy metabolism in the symbionts [43]. In rare cases (~0.5% of observed cells), a host cell was found to hold two symbionts with substantially differing levels of LPO; with one symbiont having a metabolic profile similar to an ex-symbiont (see supplementary Fig. S5c). The low occurrence of these dual LPO profile cells may be explained by the host cell, in detecting a shift in the physiology of one symbiont—or being induced by the symbiont, rapidly expels that symbiont, making this dual scenario short-lived and thereby challenging to capture. Alternatively,it may be that in general, multiple algal cells within a shared host cell will more commonly be in the same physiological state. At this time, however, it is unknown whether the host can selectively expel individual symbionts.

The role, if any, of LPO in coral symbionts is currently unknown. Controlled lipid peroxidation via enzymes such as lipoxygenase (LOX) and cyclooxygenase (COX) has been shown to help mobilise lipid stores in plant embryos and cotyledons by enabling access to the storage ester lipids in lipid bodies, thus freeing fatty acids that may subsequently be metabolised through *β*-oxidation in the mitochondria [44–46]. As such, LPO could facilitate the release of carbon from the lipid bodies for transfer to the host during active symbiosis. Also, it has been suggested recently that symbiont-derived oxylipins, important signalling molecules generated from enzymatic peroxidation of polyunsaturated fatty acids, may play a role in the maintenance of the coral symbiosis by directly triggering changes in host gene expression [47]. However, any such links to LPO in coral symbionts remain to be investigated.

### Lipid volume and peroxidation as indicator of symbiosis breakdown

**Fig. 2:**
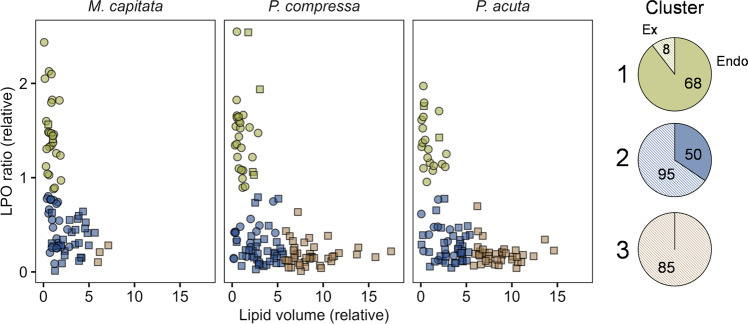
Scatterplot of LPO ratio versus lipid content relative to the mean value of endosymbiotic cells in individual coral endosymbiotic and ex-symbiotic algal cells from three species of corals (as in Fig. 1). Plot colours indicate groups identified via *Kmeans* clustering (green: cluster 1 ‘endosymbiotic’; blue: cluster 2 ‘transition’; orange: cluster 3 ‘ex-symbiotic’). Shapes indicate cell type (circle: endosymbiotic, square: ex-symbiotic). Circle diagrams show proportion of endosymbiotic (endo) vs ex-symbiotic (ex) algal cells within each cluster; numbers indicate total count of cells of each cell type.

Distinct grouping of algal cell lipid status was confirmed by cluster analysis in which three clusters were detected, with percentage distribution of (endo:ex) 90:10, 34:66 and 0:100 within clusters 1, 2 and 3, respectively (green, blue and red; Fig. 2). Based on the observed grouping, we hypothesise that cluster #1 mainly represent active endosymbiotic algae and cluster #3 represent expelled, yet physiologically active cells, whereas cluster #2 consists of cells that are either dying (low lipid content and low LPO) or that are in the intermediate physiological state that exists just before or after expulsion.

**Fig. 3:**
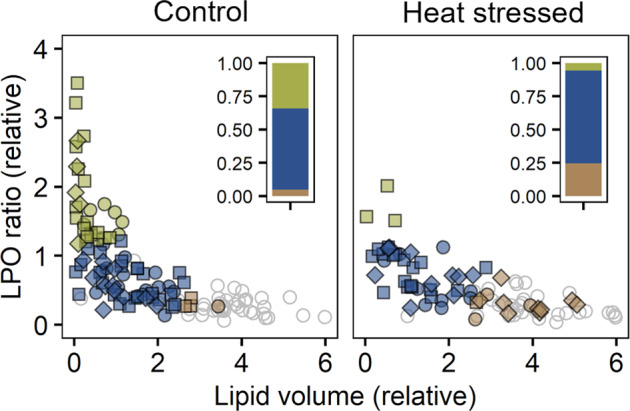
Scatterplots of LPO ratio versus lipid volume relative to the mean value of endosymbiotic cells in individual coral endosymbiotic and ex-symbiotic algal cells from control and heat-treated colonies of *Pocillopora acuta* (*n* = 3). Shapes indicate colony replicate. Raw data from Nielsen et al. [33]. Clusters identified and coloured as for Fig. 2. Only endosymbiotic cells are coloured, with ex-symbiotic cells shown as grey, open symbols. Bar graphs show the proportion of endosymbiotic cells in each cluster.

To assess the validity of these assumptions, we analysed data obtained from an earlier heat stress experiment on *Pocillopora acuta* [33]. These data showed the same relationship between LPO and lipid content as observed in the present study (see Supplementary Fig. S6 and associated statistics), where total lipid volume per cell was greater in ex-symbionts while LPO ratios were significantly lower, further confirming the constancy of this pattern. Interestingly, LPO did not increase with heat stress, supporting the hypothesis that the difference in LPO between endosymbionts and ex-symbionts is not caused by stress-related ROS production, at least not in *P. acuta*. Using cluster analysis on individual cells we found that heat stress consistently reduced the number of endosymbiotic cells within the lipid profile of cluster 1 (Fig. 3, also see Supplementary Fig. S7 for plot of individual replicate colonies), strongly supporting the hypothesis that cluster 1 represents healthy or non-stressed cells *in-symbio*. In a recent study, we found that heat stress resulted in a reduction in the proportion of key proteins linked to energy production in the symbionts of the coral *Acropora milllepora* [43]. A lowering of energy metabolism is likely to also reduce general ROS production, which could explain the reduction in the proportion of high LPO endosymbionts observed here. These data also indicate that healthy endosymbionts are more metabolically active and therefore may explain the drop in LPO when going from endosymbiotic to ex-symbiotic state even without the involvement of environmental stress. While only heat stress was tested as a driver of the shift in LPO and lipid content of endosymbiotic cells here, the clear difference observed between endo- and ex-symbiotic cells in healthy corals suggest that the response is likely to be of a general nature no matter the cause for metabolic change. This, however, remains to be investigated. The importance of lipids in the coral symbiosis was highlighted in an earlier study on the coral *Euphyllia glabrescens* [48], where the extent of accumulation of lipid bodies within the host gastrodermal cells and endosymbionts was shown to correlate with the apparent health of the symbiosis, as modulated by heat stress. Our study supports and furthers these observations by showing that the pattern of lipid body accumulation and LPO in endosymbionts can be used as an indicator for the imminent collapse of the symbiotic interactions at the cellular level. The reason for the accumulation of lipids up to and/or after the breakdown of the symbiosis is unknown. However, previous studies have shown that the presence of an unknown host release factor causes the algae to increase their production of glycerol by diverting surplus carbon away from storage compounds [49–51]. If the symbiotic relationship is breaking down, the disappearance of this host release factor could prompt the symbiont to accumulate surplus carbon as lipid instead. A recent study has suggested that leading up to the collapse of the symbiosis, increased nitrogen supply from the host to the symbiont causes a shift in the metabolic priority of the symbiont towards growth and cell division [52]. With an increase in nitrogen supply, it is conceivable that the resulting decrease in C:N ratio prompts the symbiont to hold on to excess carbon which may be useful for continued growth, causing an increase in the amount of carbon stored as lipid bodies within the cell. On the other hand, algal cells including free-living *Symbiodinium* has also been shown to increase lipid storage upon experiencing nitrogen limitation [53], which would likely come about after expulsion from the host cell. Whichever way around, the increase in lipid bodies within the algal cell seems to point to a loss of symbiotic status and supports previous observations of a link between symbiosis and lipogenesis in the algae.

Based on the findings presented, we propose that coral endosymbionts exhibit high levels of lipid body LPO compared to expelled symbionts due to comparatively higher metabolic activity and lower lipid stores. Contrary to expectation, LPO was not an indicator of stress under the bleaching conditions tested, presenting yet another example of coral bleaching that occurs independently of excess ROS production. Our results suggest that lowered LPO together with an increase in lipid stores is a robust indicator of changes in the metabolic profile of coral endosymbionts and a useful cellular indicator of the imminent breakdown of the coral symbiosis. In verifying this pattern across three distantly related coral species, we show that these observations have broad phylogenetic relevance, and thereby may represent a generally applicable metabolic marker for characterising the state of the coral symbiosis. The combination of single-cell measurements and cluster analysis revealed a small but distinct shift in the proportion of cells of a specific metabolic profile in *P. acuta* that could be attributed to heat stress. Such subtle effect is easily overlooked in bulk tissue analyses and indeed could not be detected in mean colony responses, highlighting the strength and importance of single-cell work for revealing important physiological mechanisms of coral-algal symbiosis. Together with the recent advances in tissue culture techniques for growing and maintaining individual symbiotic and non-symbiotic coral cells [7], this new marker provides an exciting opportunity for the investigation of mechanisms underlying the maintenance and breakdown of the coral symbiosis.

## Supplementary information

Supplemental material

Main data file

## Data Availability

The datasets generated during and/or analysed during the current study are available from the corresponding author on reasonable request. Data for figures in the manuscript are included in supplementary materials data.xlsx.
