# Supplementary material for: Lipid stores reveal the state of the coral-algae symbiosis at the single-cell level
Source: ISME Commun. 2023 Apr 4;3:29. doi: 10.1038/s43705-023-00234-8 (PMC10073229; doi:10.1038/s43705-023-00234-8)
Supplement: Supplementary file 1 — Supplemental material [file 43705_2023_234_MOESM1_ESM.pdf]

Supplementary Information for

Lipid stores reveal the state of the coral-algae symbiosis at the single cell level

Daniel Aagren Nielsen*, Katherina Petrou

Corresponding author:

Daniel Aagren Nielsen

Email: danielaagrennielsen@gmail.com

This PDF file includes:

Supplementary text

Tables S1 to S3

Figures S1 to S7

SI References

Supplementary Materials and Methods

General image analysis (detailed description)

All images were analysed in ImageJ/Fiji (1, 2). Firstly, individual endosymbiotic host cells or free algal cells were manually cut out of each confocal image and saved as individual image stacks for subsequent processing. This step ensured minimum amount of background interference. For each layer of each image stack (15-20 layers per stack), algal cells (endosymbiotic, ex-symbiotic or expelled) were manually identified based on chlorophyll auto-fluorescence and delineated using the Cell Magic Wand (v1.0) tool for ImageJ and the regions of interest (ROI) saved. Quantification and fluorescence measurements of lipid bodies within each cell was carried out using a custom macro, processing the images using the following steps: (1) removal of background fluorescence from all layers of the image by subtracting the average background for each layer (measured in all 4 corners) + 1 standard deviation to reduce influence of pixel noise, (2) detection of fluorescent lipid bodies within regions of interest (symbiont cells) and outside regions of interest (host cell) using the 'find peaks' function, with outlining of each lipid body based on a fixed intensity cut-off, (3) measurement of each lipid region in each fluorescence layer representing oxidised and reduced lipid fluorescence, respectively, and 4) saving the measurements of each lipid body to file (size, location and fluorescence intensities). To ensure fixed thresholds and sampling parameters, macro settings were kept constant for all cell images across type and treatment within a dataset. However, due to changes to microscope laser intensities at each wavelength between projects (present study and Nielsen *et al*. 2018), macro intensity cut-off settings were optimised for images from the two projects independently, to ensure optimal detection of lipid bodies. Additionally, data from the two projects were analysed, interpreted, and presented separately. All extracted image data were analysed using R (3). The fluorescence ratio of each lipid body was calculated and the average fluorescence ratio of all lipid bodies within each cell presented. An estimate for total lipid body volume per cell was generated as the sum of the area of all lipid body ROIs within a cell multiplied by the image layer focal thickness (~1 µm). For endosymbiotic cells, the average of the two cells within one endoderm cell are presented as one measurement. The large, round and highly fluorescent inclusion bodies sometimes found in symbiont cells were excluded from the dataset using fixed size range and fluorescence ratio cut-offs based on manual curation. For further details on this processing please refer to SI in Nielsen *et al.* 2018 (4).

Assessment of symbiosome fluorescence

To assess any relationship between the presence of a symbiosome membrane and the lipid profile of the algal cell, we used a symbiosome dye (MDY-64) (5) in conjunction with the LPO ratio dye. Unfortunately, due to fluorescence bleed from the symbiosome channel into the channel for oxidated lipid, we could not assess the relationship between LPO ratio and the symbiosome membrane. Additionally, because of presence of auto-fluorescence in the symbiosome channel, a clear absence/presence of a symbiosome membrane could not be rendered. Instead, we present the absolute symbiosome and reduced lipid fluorescence (limited to the algal symbiont) taken from a single image within the image stack approximately half-way through the algal cell (largest diameter), together with the designation of cell type based on observation of a host cell membrane around the algal cell and whether one or two algal cells were present inside the same membrane. The relationship between lipid content and symbiosome fluorescence was assessed using a linear mixed effect model with colony as random effect to account for the repeated nature of the data (multiple cell types from the same colony).

Supplementary Results and Discussion

Ex-symbiotic cells are different from single and double endosymbiotic cells

To assess whether ex-symbiotic cells were different from single endosymbiotic and whether single endosymbiotic cells were different from double endosymbiotic cells, we analysed and compared lipid content and lipid peroxidation ratio for each cell type from all three species of corals (Figure S1). In all species, there was a significant effect of cell type on lipid volume (M. *capitata*: F(4) = 8.4395, $P = 0.0367$; ; *P. compressa*: F(6) = 262.19, $P < 0.0001$; *P. acuta*: F(6) = 43.829, $P = 0.00026$) (Fig S1a) and LPO ratio (M. *capitata*: F(4) = 47.723, $P = 0.0016$; ; *P. compressa*: F(6) = 53.23, $P = 0.00015$; *P. acuta*: F(6) = 37.156, $P = 0.00042$) (Fig S1b). Tukeys post-hoc test showed that single endosymbiotic cells were different from ex-symbiotic cells for both *P. compressa* and *P. acuta,* but not in *M. capitata*. Additionally, double endosymbiotic cells were not different from single endosymbiotic cells in *M. capitata* and *P. acuta,* but was significantly different for *P. compressa*. The differences in LPO ratio were more consistent, with both double and single endosymbiotic cells being different from ex-symbiotic cells but

not from each other. From these results, it is clear that ex-symbiotic cells are indeed different from single-endosymbiotic cells, which themselves are similar to double endosymbiotic cells.

Ex-symbiotic cells are more similar to fully expelled algal cells

To assess whether ex-symbiotic cells found inside the coral tissue but outside a host cell were similar to fully expelled cells (expelled from the coral host colony and captured in the water column), we analysed and compared endosymbiotic cells, ex-symbiotic cells and fully expelled cells from three colonies of *P. acuta* (Fig. S2). There was a significant difference in lipid content between cell types (Fig. S2a) ($F(6) = 78.137$, $P < 0.0001$), with Tukeys post-hoc test identifying differences between all cell types. However, both ex-symbiotic cells and fully expelled cells exhibited higher lipid content than endosymbiotic cells. In contrast, differences in LPO (Fig. S2b) were only observed between endosymbiotic cells and the two groups of ex- and fully expelled cells ($F(6) = 18.668$, $P = 0.0027$). These results strongly support the hypothesis presented in the main manuscript that ex-symbiotic cells are similar to fully expelled algal cells, while fully expelled cells are further advanced in the symbiotic separation, having left the coral colony as a whole, and therefore may have had longer to accumulate lipid, explaining the increased lipid content in these cells compared to ex-symbiotic cells.

Lipid content is negatively correlated with symbiosome fluorescence

To qualitatively assess a relationship between symbiotic state of the algal symbiont and the lipid content, we investigated the possibility for a correlation between the presence of a symbiosome membrane and the lipid content in the algal cell. The results show a clear negative relationship ($R^2 = 0.37$, $F(116) = 70.836$, $P < 0.0001$) between lipid content and the intensity of the symbiosome membrane fluorescence (Figure S3), where the likelihood of the presence of an actual symbiosome increases with increasing fluorescence. Additionally, a clear grouping can be seen, with all cells identified as endosymbiotic are found at the end of higher symbiosome fluorescence and lower lipid content, and single and double endosymbiotic cells group together. Several ex-symbiotic cells are found in the same area as endosymbiotic cells, suggesting a certain overlap between the two groups in the central range, but rarely at the extremes. The few ex-symbiotic cells seemingly sitting at the extreme end of the endosymbiotic cluster could be mis-classified and truly be endosymbiotic cells (it can sometimes be hard to distinguish the host membrane around the algal cell). On the other hand, no cells classified as endosymbiotic sit at the other extreme (high lipid content and low symbiosome fluorescence).

Endosymbiotic cells have elevated LPO ratios at similar lipid content

The effect of lipid content and cell type on the LPO response variable was investigated first using linear mixed effect models (using function lmer() in R package lme4) with colony as random effect. However, in all models the variance explained by the random effect was <3% of the total variance, indicating this effect was not significant and hence data were instead fitted using generalised least squares regression (gls) with maximum likelihood (ML) optimisation of estimates (Table S3). Models were compared based on Bayesian information criterion (BIC) and using the anova function. The model with the best fit ($P < 0.0001$) contained both lipid volume and cell type as predictor variables, verifying that while variation in lipid content can explain some of the variability in LPO, this relationship is different between cell types. Inclusion of species as a descriptor did not improve the model significantly and resulted in a slightly elevated BIC.

Physiological stress is not the driver of differences between endo and ex-symbiotic cells

Lipid content of endosymbionts and ex-symbionts was significantly different ($F(9) = 43.376$, $P = 0.0001$), with Tukeys post-hoc test showing differences in both control and heat-treated cells, showing that ex-symbiotic cells had greater amounts of lipid compared to their endosymbiotic counterparts (Fig. S6a). Conversely, LPO was greater in the endosymbionts compared with ex-symbionts ($F(7) = 104.781$, $P < 0.0001$) for both control and heat-treated cells (Fig. S6b). There was no effect of heat treatment in either case, indicating that metabolic stress was not the driver of the observed differences in lipid and LPO between cell types.

Supplementary tables

Table S1. Output from Linear mixed effect model using restricted maximum likelihood (REML) optimisation of parameter estimates for pairwise comparisons of lipid and LPO between double and single endosymbiotic and ex-symbiotic cells from three species of corals (Fig. S1). All P values adjusted for multiple comparisons using the Tukeys method.

Species	Lipid/ LPO	Comparison	Estimate	SE	df	T ratio	P value	Sig. level
M.cap	Lipid	double_endo vs single_endo	-0.0555	0.449	4	-0.124	0.9916	ns
M.cap	Lipid	double_endo vs ex-symbiont	-3.618	0.449	4	-3.618	0.0477	*
M.cap	Lipid	single_endo vs ex-symbiont	-3.495	0.449	4	-3.495	0.0531	ns
M.cap	LPO	double_endo vs single_endo	-0.148	0.0496	4	-2.981	0.0849	ns
M.cap	LPO	double_endo vs ex-symbiont	0.326	0.0496	4	6.566	0.0061	**
M.cap	LPO	single_endo vs ex-symbiont	0.474	0.0496	4	9.548	0.0015	**
P.com	Lipid	double_endo vs single_endo	-0.888	0.24	4	-3.702	0.0444	*
P.com	Lipid	double_endo vs ex-symbiont	-5.13	0.24	4	-21.421	0.0001	***
P.com	Lipid	single_endo vs ex-symbiont	-4.250	0.24	4	-17.719	0.0001	***
P.com	LPO	double_endo vs single_endo	0.146	0.0648	4	2.260	0.1738	ns
P.com	LPO	double_endo vs ex-symbiont	0.638	0.0648	4	9.849	0.0013	**
P.com	LPO	single_endo vs ex-symbiont	0.492	0.0648	4	7.589	0.0036	**
P.acuta	Lipid	double_endo vs single_endo	0.115	0.852	6	0.135	0.9900	ns
P.acuta	Lipid	double_endo vs ex-symbiont	-6.848	0.852	6	-8.040	0.0005	***
P.acuta	Lipid	single_endo vs ex-symbiont	-6.963	0.852	6	-8.175	0.0004	***
P.acuta	LPO	double_endo vs single_endo	0.0315	0.0934	4	0.337	0.9403	ns
P.acuta	LPO	double_endo vs ex-symbiont	0.7126	0.0934	4	7.628	0.0035	**
P.acuta	LPO	single_endo vs ex-symbiont	0.6812	0.0934	4	7.291	0.0042	**

Table S2. Output from Linear mixed effect model using restricted maximum likelihood (REML) optimisation of parameter estimates for pairwise comparisons of lipid and LPO between double endosymbiotic, ex-symbiotic and fully expelled cells from *P. acuta* (Fig. S2). All P-values are adjusted for multiple comparisons using the Tukeys method.

Lipid / LPO	Comparison	Estimate	SE	df	T ratio	P value	Sig. level
Lipid	double_endo vs ex-symbiont	-0.934	0.119	4	-7.828	0.0032	**
Lipid	double_endo vs expelled	-1.474	0.119	4	-12.355	0.0006	***
Lipid	ex-symbiont vs expelled	-0.540	0.119	4	-4.527	0.0230	*
LPO	double_endo vs ex-symbiont	0.614	0.115	4	5.356	0.0128	*
LPO	double_endo vs expelled	0.599	0.115	4	5.225	0.014	*
LPO	ex-symbiont vs expelled	-0.015	0.115	4	-0.130	0.9907	ns

Table S3. Output of Generalised Least Squares linear regression models using maximum likelihood (ML) optimisation of parameter estimates for the relationship between lipid and LPO content in two different cell types (Fig S4). The lowest BIC was obtained for model 3 which also was evaluated as the best model when compared using an ANOVA ($P < 0.0001$). df: degrees of freedom, BIC: Bayesian information criterion (smaller value indicates better model).

#	Model	df	BIC
1	Log10(LPO_ratio) ~ sqrt(lipid_volume)	333	309.1258
2	Log10(LPO_ratio) ~ cell_type	333	263.4696
3	Log10(LPO_ratio) ~ sqrt(lipid_volume) + cell_type	333	233.2232
4	Log10(LPO_ratio) ~ sqrt(lipid_volume) + cell_type + Species	333	241.2354

Supplementary Figures

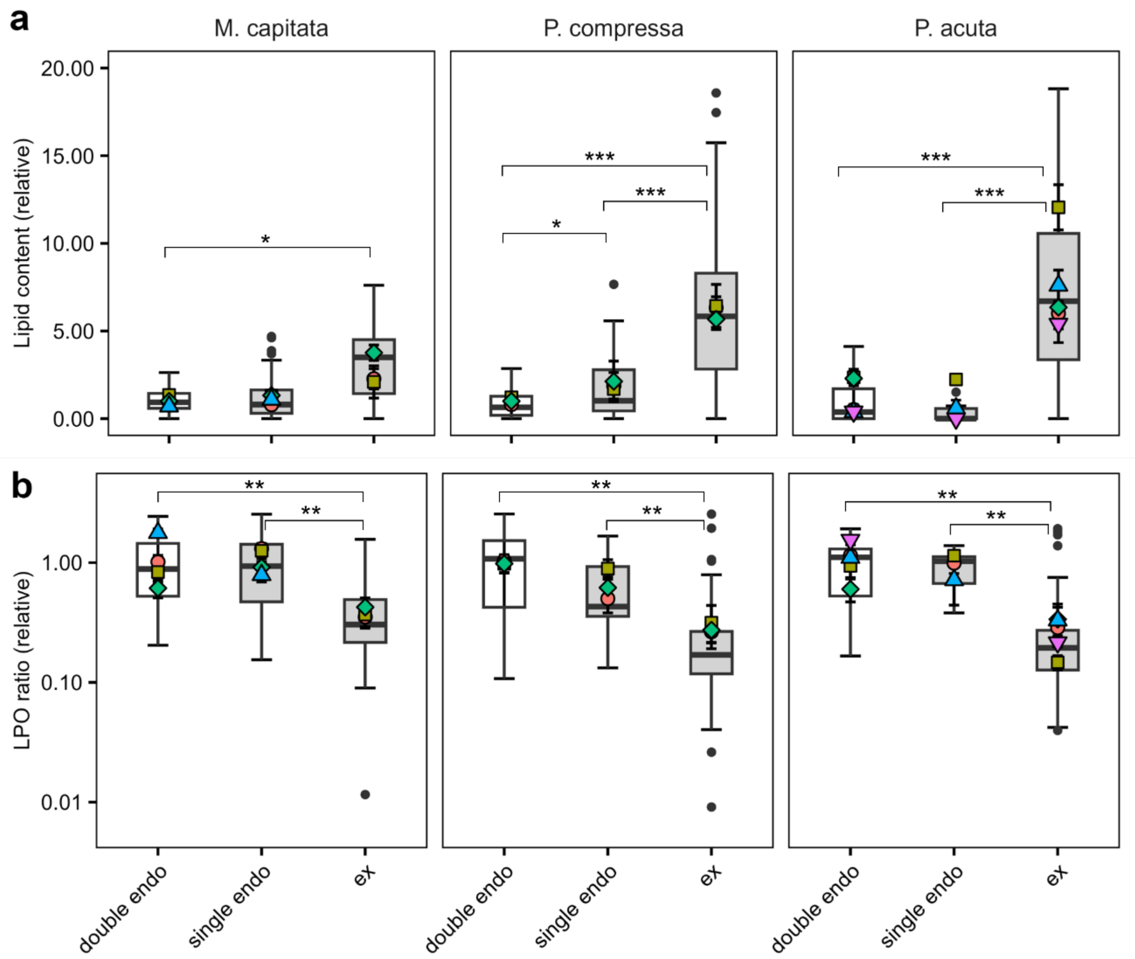

Figure S1. Comparison of lipid volume (a) and lipid peroxidation ratio (LPO) ratio (b) between double and single endosymbiotic cells and ex-symbionts (ex) in three species of hard corals. Coloured symbols represent coral colonies. Errors bars (on symbols) indicate 1 SE. Stars indicate significance level for comparison of groups using mixed effect models with colony as random effect and Tukeys post-hoc for pairwise analyses and *P*-adjustment (* $P < 0.05$; ** $P < 0.01$, *** $P < 0.001$).

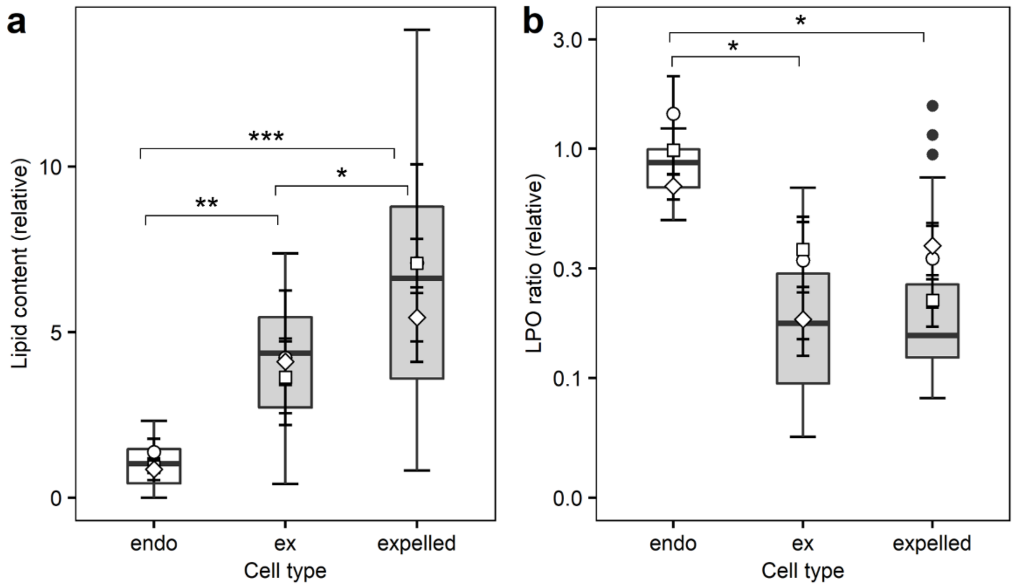

Figure S2. Boxplots of lipid volume and lipid peroxidation (LPO) ratio of "endo" -symbionts (white) and non-host associated "ex" -symbiotic (algal cells without host membrane located inside coral tissue) and "expelled" algal cells (grey) from the hard coral *Pocillopora acuta* overlaid with mean colony value. The similarity between ex-symbiotic cells and expelled cells show that these are comparable in physiology. Errors bars (on symbols) indicate 1 SE (n=3). Stars indicate significance level for comparison of groups using mixed effect models with colony as random effect and Tukeys post-hoc for pairwise analyses and *P*-adjustment (* $P < 0.05$; ** $P < 0.01$, *** $P < 0.001$).

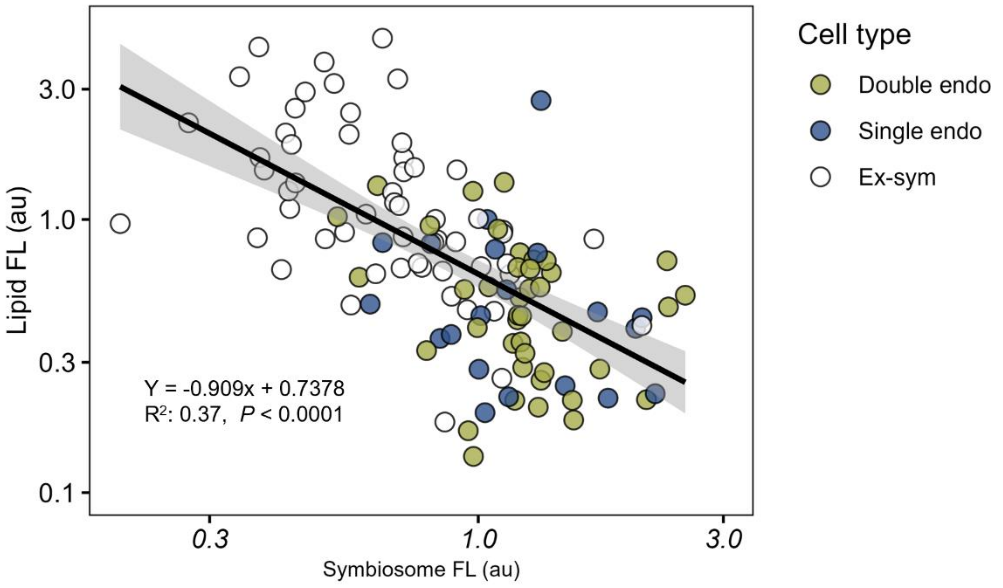

Figure S3. Relationship between lipid content and symbiosome membrane fluorescence in endosymbiotic and ex-symbiotic algal cells. While ex-symbiotic algae do not have a symbiosome membrane some auto-fluorescence was observed that made a presence-absence distinction impossible. Open circles are cells for which no host membrane could be observed (designated ex-symbiotic). Coloured circles are cells positively identified as double (green) or single (blue) endosymbiotic algal cells via presence of clear host membrane. Line represents linear regression across all cell types (R^2 = 0.37, $P < 0.0001$). The strong relationship indicates that when a symbiosome is present (cell is endosymbiotic) cells are more likely to have low lipid content.

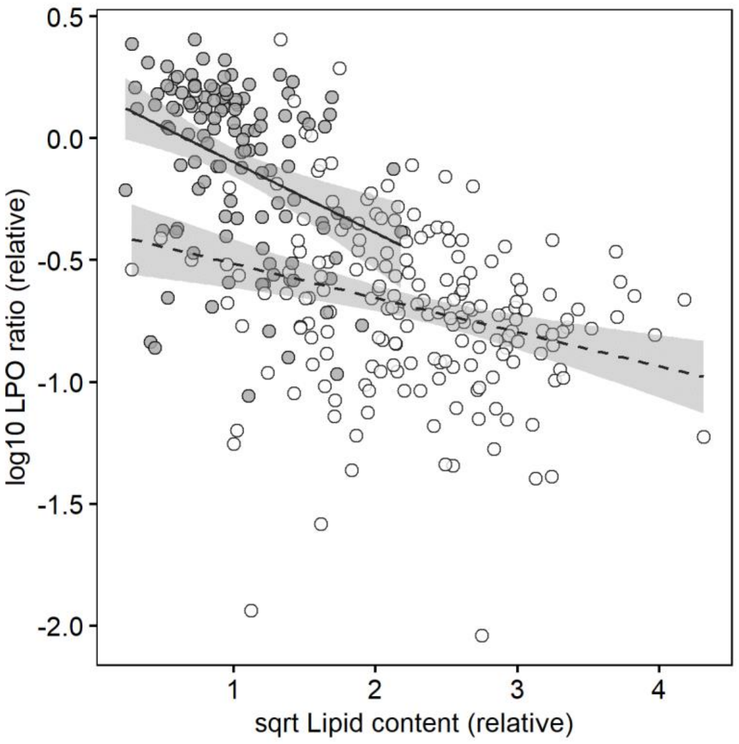

Figure S4. Lipid peroxidation ratio (LPO) versus lipid content of endosymbiotic (grey) and ex-symbiotic (white) algal cells from three species of hard corals (*Montipora capitata, Porites compressa, Pocillopora acuta*). Linear regressions show the general trend of the relationship between LPO ratio and lipid content in the two types of cells. A generalised least squares regression model with both lipid volume and cell type as explanatory variables produced the best fit to the data. Endosymbiotic cells generally exhibit higher LPO ratio at low lipid volume compared to ex-symbiotic cells.

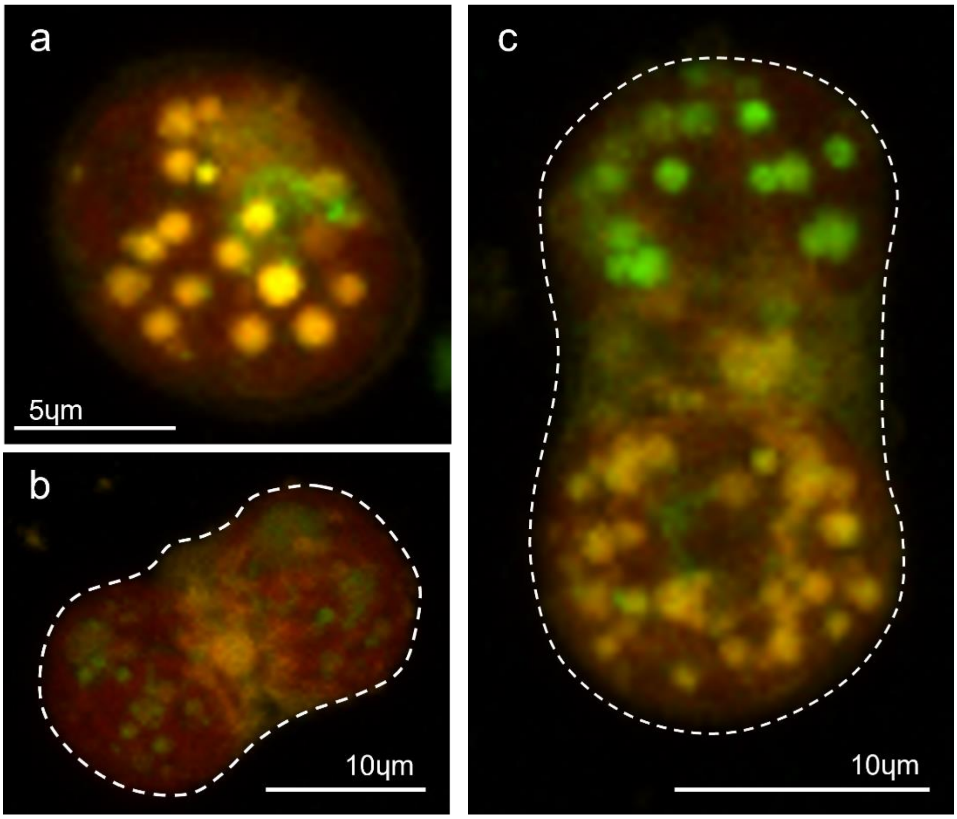

Figure S5. Example images (maximum projection) of (a) ex-symbiotic algal cell with large, reduced lipid bodies (yellow); (b) endosymbiotic host cell with two agal symbionts displaying highly oxidised lipid bodies (green); (c) rare scenario of host cell with two endosymbiotic algal cells in different physiological stages with one displaying highly oxidised (green) lipid bodies (top) and the other reduced (yellow) lipid bodies but higher total volume of lipid bodies. Host cells containing agal cells in two different stages were only observed in ~0.5% of all cells, suggesting algal cells with sub-optimal physiologies are quickly released from the host cell and/or that algal cells within a host cell is more commonly in the same physiological state. Red colour represents autofluorescence from algal chlorophyll *a*. White stippled line indicates location of host cell membrane.

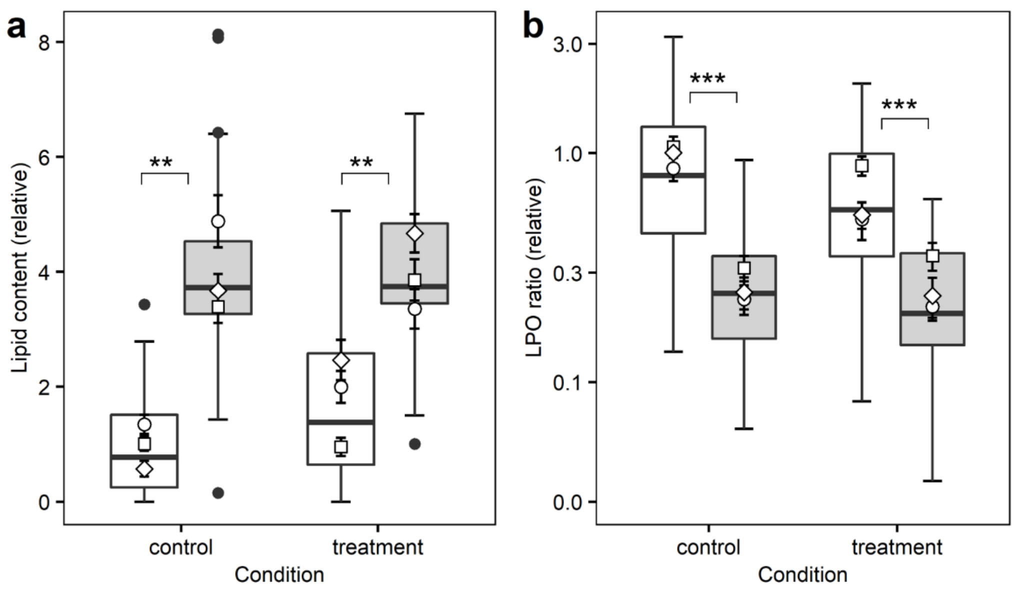

Figure S6. Boxplots of lipid volume and lipid peroxidation (LPO) ratio of endosymbionts (white) and non-host associated (ex-symbiotic) algal cells (grey) of the hard coral *Pocillopora acuta* at control and elevated temperature conditions (see method description in main paper). (a) lipid volume relative to the mean of their respective endosymbionts, overlaid with mean colony value. (b) Lipid peroxidation ratio relative to the mean of their respective endosymbionts, overlaid with mean colony value. Errors bars (on symbols) indicate 1 SE (n=3). Stars indicate significance level for comparison of groups using mixed effect models with colony as random effect and Tukeys post-hoc for pairwise analyses and P-adjustment (* $P < 0.05$; ** $P < 0.01$, *** $P < 0.001$).

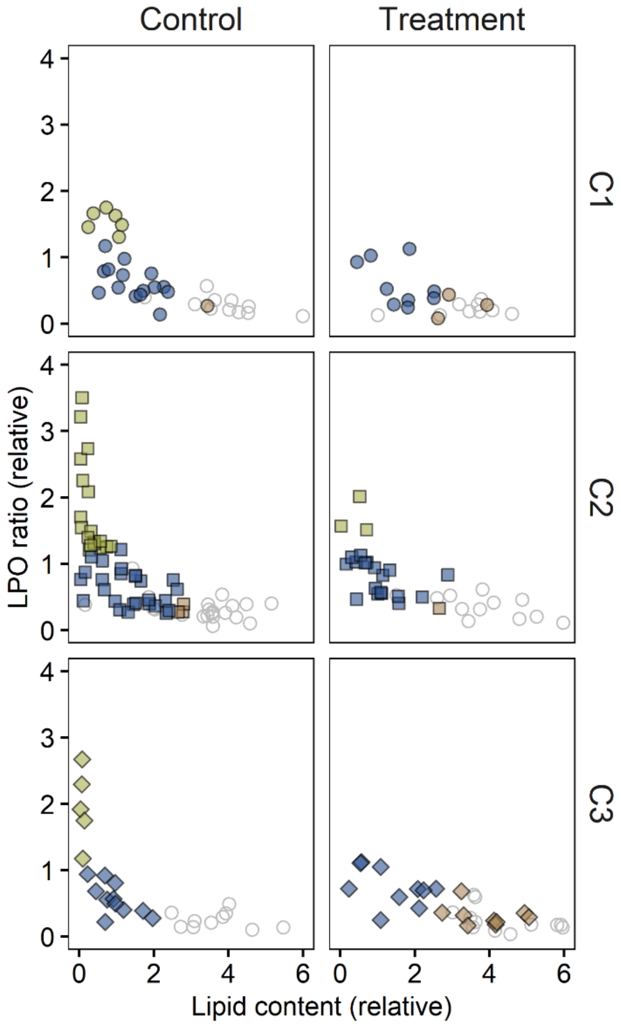

Figure S7. Scatterplots of LPO ratio versus lipid content relative to the mean value of endosymbiotic cells in individual coral endosymbiotic and ex-symbiotic algal cells for each colony replicate (C1-3) from control and heat-treated specimens of *Pocillopora acuta*. Colours delineate three clusters identified via *Kmeans* clustering (green: cluster 1 "endosymbiotic"; blue: cluster 2 "transition"; orange: cluster 3 "ex-symbiotic"). Only endosymbiotic cells are coloured with ex-symbiotic cells shown as grey, open symbols. In all colonies the number of endosymbiotic cells in cluster 1 (green) is reduced with heat treatment, indicating these cells are primarily affected by heat stress and therefore likely to be healthy endosymbiotic cells under control conditions. Raw data obtained from Nielsen *et al*. 2018 and reanalysed for the purpose of the present study.

SI References

1. Schneider CA, Rasbnad WS, Eliceiri KW. NIH Image to ImageJ: 25 years of image analysis. Nature Methods. 2012;9(7):671-9.

2. Schindelin J, Arganda-Carreras I, Frise E, Kaynig V, Longair M, Pietzsch T, et al. Fiji: an open-source platform for biological-image analysis. Nature methods. 2012;9(7):676.

3. Team RC. R: A language and environment for statistical computing. R Foundation for Statistical Computing, Vienna, Austria. 2021.

4. Nielsen DA, Petrou K, Gates RD. Coral bleaching from a single cell perspective. Isme Journal. 2018;12(6):1558-67.

5. Trautman DA, Hinde R, Cole L, Grant A, Quinnell R. Visualisation of the symbiosome membrane surrounding Cnidarian algal cells. Symbiosis. 2002;32(2):133-45.
